# It takes two to tango: Plant hosts influence bacterial effector function through post-translational modifications

**DOI:** 10.1093/plcell/koae059

**Published:** 2024-02-27

**Authors:** Bradley Laflamme

**Affiliations:** Assistant Features Editor, The Plant Cell, American Society of Plant Biologists; Department of Molecular Genetics, University of Toronto, Toronto, ON M5S 1A1, Canada

Many bacterial phytopathogens secrete virulence proteins (often termed effectors) directly into plant cells to aid in infecting and colonizing host tissues (Cunnac et al. 2011). While certain effectors have well-established virulence functions inside of the plant cell—typically involving interference with host immune mechanisms—we have a paler understanding of how the host might influence effector activities. After all, eukaryotic cells are wired with organelles and biological processes that prokaryotes do not possess. However, prokaryotic pathogens, despite lacking these cellular mechanisms, display an intriguing ability to leverage and exploit eukaryotic features during infection. Thus, it is exciting to consider how coevolution has driven prokaryotic pathogens to integrate eukaryotic tools into their infection strategy.

In this issue, Li and colleagues (Li et al. 2024) investigated the contribution of SUMOylation (SUMO = Small Ubiquitin-related MOdifier), a common post-translational modification (PTM) in eukaryotes, to the function of effectors from the bacterial pathogen *Pseudomonas syringae*, a model bacterial phytopathogen with a well-cataloged effector repertoire. By performing an in vitro SUMOylation screen in *Escherichia coli* engineered to express Arabidopsis SUMOylation machinery, the group found that 16 out of 36 *P. syringae* effectors could be SUMOylated. This screen thus suggested that almost one-half of the tested bacterial effectors—spanning a broad range of biochemical functions and in planta targets—could potentially serve as substrates for PTMs once inside the plant cell. The group then focused on 2 SUMOylated effectors with well-established virulence activities, HopB1 and HopG1, to characterize the impact of their PTMs on *P. syringae* pathogenesis—after, of course, confirming that both effectors were SUMOylated in planta.

HopB1 is a serine protease that cleaves the Arabidopsis co-receptor BAK1, inducing a host immune response that results in programmed cell death (Li et al. 2016). Remarkably, a HopB1 mutant deficient in 2 SUMOylation sites was found to be more effective at cleaving this receptor, ultimately triggering a more robust cell death response than the wild-type allele and having a detrimental impact on virulence when expressed in *P. syringae*. HopG1 is an effector that localizes to the plant mitochondria, where it induces mitochondrial dysfunction and oxidative stress (Block et al. 2010). A SUMOylation-deficient mutant of HopG1 showed reduced virulence activities, resulting in a lower accumulation of reactive oxygen species and a higher number of functional plant mitochondria per cell. Thus, SUMOylation of both HopB1 and HopG1 appears to benefit *P. syringae*, resulting in increased effector virulence (Fig.).

**Figure.**
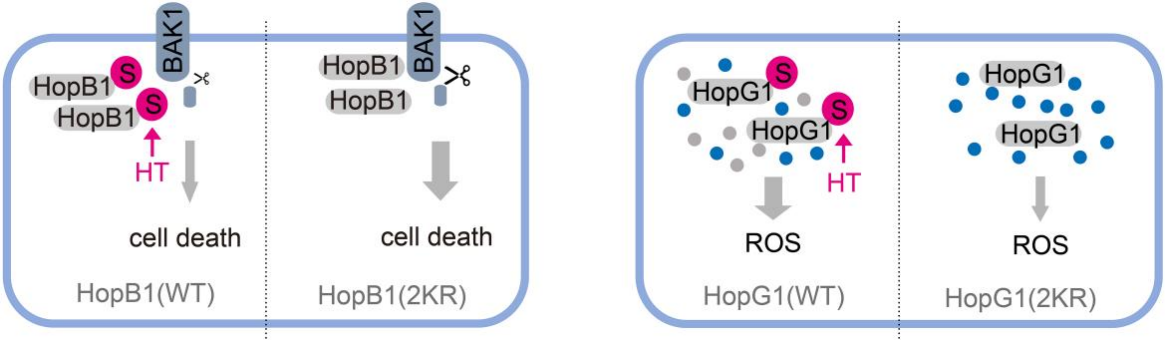
A proposed model for SUMOylation of HopB1 and HopG1 in plant infection. A SUMOylation-deficient mutant (2KR) of HopB1 triggers an amplified cell death response by cleaving the BAK1 transmembrane receptor that is detrimental to *P. syringae* virulence. A similar 2KR mutant of HopG1 fails to damage mitochondria (represented by green dots for active mitochondria and grey dots for inactive), ultimately leading to a reduced production of reactive oxygen species (ROS). S: SUMO; HT: high temperature. Reproduced from Figure 4I.

The authors simultaneously investigated the transcriptomic responses of plants to both the wild-type and SUMOylation-deficient alleles of both HopB1 and HopG1. Their analyses demonstrated that SUMOylation dampens the induction of immune-related genes by HopB1 and improves the capacity of HopG1 to positively regulate genes encoding negative regulators of jasmonic acid signaling, thus showing that the observed benefits of SUMOylations to pathogen virulence can be reflected in the host transcriptome. Additionally, the group explored the impact of heat stress on effector function, as SUMOylation of HopB1 and HopG1 was increased at higher temperatures. Remarkably, both effectors had a greater requirement for SUMOylation at higher temperatures: the immune-activating properties of the HopB1 SUMOylation-deficient mutant were exacerbated at higher temperatures, leading to reduced pathogen virulence, while the SUMOylation-deficient mutant of HopG1 lost much of its virulence outputs at higher temperatures. Thus, in a warming climate, the pathogen's cooption of host PTMs may be of greater importance to establishing infection.

Li and colleagues provide strong evidence for SUMOylation as an underacknowledged factor that regulates the interactions between plants and phytopathogenic bacteria, and their findings on the effects of heat stress on effector SUMOylation may also have implications in a warming climate. Their observations are especially interesting because bacterial effectors frequently “mimic” host proteins in function, likely to conceal their invasion of host tissues (Galán 2009). Interestingly, most of the effectors present in the “minimal functional repertoire” of *P. syringae—*that is a reduced, core set of 8 effectors that the pathogen can use to achieve virulence (Cunnac et al. 2011)—are likely SUMOylation substrates. Considering the use of SUMOylation sites by bacterial pathogens from an evolutionary perspective (as the authors encourage us to do), we might opine that the adoption of eukaryotic sequence motifs represents a key step in the development of bacterial pathogenesis. In this case, the plant can only be so upset about getting infected—after all, the bacterium learned some of its tricks from its host.

## Dive Curated Terms

The following phenotypic, genotypic, and functional terms are of significance to the work described in this paper:

PTM AmiGo: PO:0005039

BAK1 Gramene: AT4G33430
BAK1 Araport: AT4G33430
